# The G-Protein-Coupled Estrogen Receptor Selective Agonist G-1 Attenuates Cell Viability and Migration in High-Grade Serous Ovarian Cancer Cell Lines

**DOI:** 10.3390/ijms25126499

**Published:** 2024-06-13

**Authors:** Donia Hanafi, Rob U. Onyenwoke, K. Sean Kimbro

**Affiliations:** 1 Julius L. Chambers Biomedical/Biotechnology Research Institute, North Carolina Central University, Durham, NC 27707, USA; dhanafi2023@gmail.com; 2 Department of Biological and Biomedical Sciences, North Carolina Central University, Durham, NC 27707, USA; ronyenwo@nccu.edu; 3 Biomanufacturing Research Institute and Technology Enterprise (BRITE), North Carolina Central University, Durham, NC 27707, USA; 4 Department of Microbiology, Biochemistry and Immunology, Morehouse School of Medicine (MSM), Atlanta, GA 30310, USA

**Keywords:** GPER receptor, therapeutic, fallopian tubes, ovarian cancer

## Abstract

The G-protein-coupled estrogen receptor (GPER; G-protein-coupled estrogen receptor 30, also known as GPR30) is a novel estrogen receptor and has emerged as a promising target for ovarian cancer. GPER, a seven-transmembrane receptor, suppresses cellular viability and migration in studied ovarian cancer cells. However, its impact on the fallopian tube, which is the potential origin of high-grade serous (HGSC) ovarian cancer, has not been addressed. This study was conducted to evaluate the relationship of GPER, ovarian cancer subtypes, i.e., high-grade serous cell lines (OV90 and OVCAR420), as well as the cell type that is the potential origin of HGSC ovarian cancer (i.e., the fallopian tube cell line FT190). The selective ligand assessed here is the agonist G-1, which was utilized in an in vitro study to characterize its effects on cellular viability and migration. As a result, this study has addressed the effect of a specific GPER agonist on cell viability, providing a better understanding of the effects of this compound on our diverse group of studied cell lines. Strikingly, attenuated cell proliferation and migration behaviors were observed in the presence of G-1. Thus, our in vitro study reveals the impact of the origin of HGSC ovarian cancers and highlights the GPER agonist G-1 as a potential therapy for ovarian cancer.

## 1. Introduction

The most lethal gynecologic carcinoma is ovarian cancer. This cancer has a 5-year survival rate of ~45%, making it the fourth most common cancer with regard to mortality among Western nations [1,2,3]. The identification of molecular targets could aid in developing pharmacologic therapies to improve the prognosis for ovarian cancer [4]. GPER expression is known to be significantly lower in ovarian cancer tissue compared to benign and low-malignant ovarian tumors, indicating its potential role as a biomarker for disease progression and a potential molecular target for ovarian cancer [5]. The novel GPER is a 7-transmembrane G-protein-coupled receptor [6]. Until the discovery of GPER agonists and antagonists, it was challenging to determine the mechanisms through which GPER mediates its estrogen activity because estrogens are ligands for both GPER and estrogen receptors (ERs) [7,8,9]. Selective GPER agonists and antagonists have recently been discovered to differentiate GPER-mediated estrogen activity from that of ER [10,11,12]. Consequently, G-1 (1-[4-(6-bromobenzo[1,3]dioxol-5yl)-3A,4,5,9B,tetrahydro-3H-cyclopenta[c]quinolin-8-yl]-ethanone) has more recently been demonstrated to be a GPER agonist [10,13,14]. Previous studies have shown that G-1 is a high-affinity, non-steroidal and selective GPER agonist [10,11]. The G-1 agonist is substantially selective for GPER, but not ER α or ER β, making it feasible to investigate GPER’s mechanism of action in various cancers [10,11]. 

Hence, the discovery of the novel GPER-specific ligand G-1 has opened up new avenues for exploring the effects of GPER on the proliferation and migration of ovarian cancer cells [9,15]. Researchers have previously utilized G-1, a selective agonist of GPER, to investigate its effects on proliferation in OVCAR3 (a high-grade serous cancer, HGSC, with HGSC defined as a fast-growing and rapidly spreading serous cancer that is the most common type of serous ovarian cancer) and SKOV3 (a non-high-grade serous cancer, non-HGSC, with non-HGSC being rarer and causing slow-growing tumors) cells [5,16]. These findings revealed that G-1 could effectively suppress proliferation in OVCAR3 and SKOV3 cells via inhibiting cell cycle progression at the G2/M phase and by promoting caspase-dependent apoptosis [5,16]. In another study, IGROV-1 and SKOV-3, non-HGSC cell lines, were used to investigate the effects of G-1 on ovarian cancer cells [9]. The results illustrated that G-1 was able to suppress proliferation and induce apoptosis in these cells by blocking tubulin polymerization, a process crucial for cell division [9]. Additionally, the G-1 agonist has previously been suggested to have an anti-proliferative activity by interfering with microtubules via targeting the colchicine-binding region of tubulin [17]. This result suggests that G-1 may hold promise as a potential therapeutic agent for ovarian cancer treatment by targeting tubulin dynamics [9]. In a recent study, it was also observed that treatment with G-1 (1 μM) led to increased levels of H3K4me3 and p-ERK1/2 and resulted in attenuated cell proliferation and migration in Caov3 and Caov4 cells (high-grade serous ovarian cancer (HGSOC) cell lines) [15,18]. These effects were attributed to the activation of GPER by G-1, indicating a potential regulatory role of GPER in modulating cell behavior in ovarian cancer cells [15], and also indicate that compounds with interesting anti-proliferative activities should also potentially be investigated for an anti-migratory activity [19,20]. Indeed, the anti-migratory properties of new and emerging compounds are likely to be an important feature, which will ultimately allow for the discovery of novel mechanisms of action [19,20]. This point references back to this current study in that the effects of different dosages (0.01, 0.1 and 1 μM) of G-1 agonist on GPER in HGSOC cell lines (OV90 and OVCAR420), and particularly fallopian tube cell lines (FT190), remain unclear. 

Previous studies have identified HGSC ovarian cancer cell lines such as OV90 and OVCAR420 as suitable in vitro models for HGSOC, which is based on their genomic profiles closely resembling HGSOC tumors [21,22,23]. In this study, we comprehensively investigated a range of G-1 concentrations on HGSOC and fallopian tube cell lines in an attempt to understand the impacts of GPER on migration and proliferation. Therefore, our study aims to assess the dose responsiveness of G-1 and its effects on HGSOC and fallopian tube cell line migration, which has not been thoroughly examined. 

## 2. Results

### 2.1. Effect of DMSO Concentrations on Cell Viability

**Figure 1:**
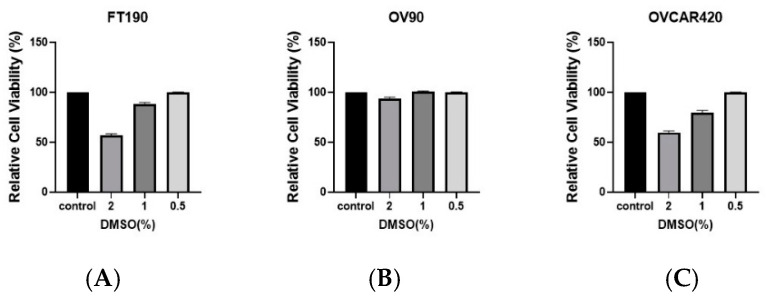
DMSO tolerance. FT190 (**A**), OV90 (**B**) and OVCAR420 (**C**) were examined against the indicated DMSO concentrations.

To assess any inhibitory effect due to the percentage (%) of dimethyl sulfoxide (DMSO; 0%, 2%, 1%, 0.5%), cell viability was determined using the OV90, OVCAR420 and FT190 cell lines along with the Cell Titer-Glo assay, as depicted in Figure 1. Treatment of OV90, OVCAR420 and FT190 with DMSO concentrations of 2% and 1% resulted in decreased cell viability while 0.5% had no effect on cell viability. 

### 2.2. Inhibitory Effect of the G-1 Agonist on Cell Viability 

**Figure 2:**
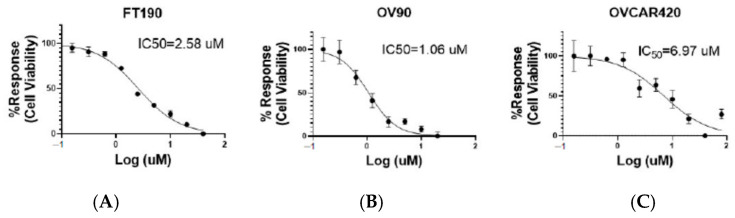
G-1 dose–response curves. G-1 concentration–response curves for FT190 (**A**), OV90 (**B**) and OVCAR420 (**C**). Estimated IC50 values were 2.58, 1.06 and 6.97 μM for FT190, OV90 and OVCAR420, respectively. Incubation was for 48 h.

To assess the potential for any inhibitory effect due to dosing with the G-1 agonist, cell viability was determined using the OV90, OVCAR420 and FT190 cell lines along with the Cell Titer-Glo assay, as depicted in Figure 2. G-1 agonist treatment of the OV90, OVCAR420 and FT190 cell lines with the G-1 agonist resulted in decreased cell viability, with varying median effective (IC_50_) concentrations observed. The OV90, OVCAR420 and FT190 IC_50_ were 1.06, 6.97 and 2.58 µM, respectively. As can be noted by their respective IC_50_, a differential sensitivity to the G-1 agonist was noted with OV90 being the most sensitive and OVCAR420 being the least sensitive. However, with all IC_50_ values being in the low µM range, the G-1 agonist proved to possess a potent inhibitory effect on all of the studied cell lines.

### 2.3. Inhibitory Effect of G-1 Agonist on Cell Migration

**Figure 3:**
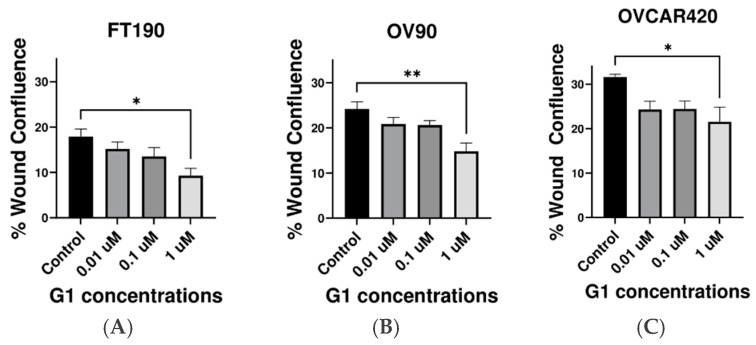
Effect of GPER on migration. (**A**–**C**) Migration assays were performed using FT190, OV90 and OVCAR420 cells treated with the G-1 agonist for 12 h. Data are presented as the mean ± SEM for three separate experiments. * *p* < 0.05 vs. control, ** *p* < 0.005 vs. control. Statistical analyses were performed using ANOVA followed by Dunnett’s post hoc test.

Cell migration is a critical step in the metastatic progression of ovarian cancer. Thus, the impact of the G-1-induced activation of GPER on cell migration was assessed using the wound healing assay. A “Wound Healing” assay (also known as a “Scratch Assay”) was conducted to better characterize the role of GPER in cell migration (Figure 3). Untreated FT190 and OV90 cells exhibited a much slower wound-healing rate as compared with untreated OVCAR420 cells. The wound healing assay revealed a substantial reduction in cell migration at 1 µM G-1 in all of the examined cell lines. Moreover, G-1 agonist at 0.01, 0.1 or 1 µM all inhibited migration in all of the studied cell lines. Thus, a 12-hour treatment with 1 μM G-1 significantly decreases migration in FT190, OV90 and OVCAR420 cells.

## 3. Discussion

In the present study, our investigation focused on studying the impact of GPER on cell viability and migration in high-grade serous ovarian cancer cell lines, as well as in a cell line originating from the fallopian tube. This was achieved through a series of experimental methods and techniques. To investigate whether GPER/GPER activity might affect the viability of ovarian cancer cells, OV90, OVCAR420 and FT190 cells were incubated with increasing concentrations of the GPER-specific agonist G-1 for 48 h. Previous studies have demonstrated that the G-1 agonist can induce cell cycle arrest at 48 h, as measured via flow cytometry [5,15]. As a result, cell viability decreases, allowing for IC_50_ values to be determined [5,15]. However, the impact of G-1 activation of GPER on OV90, OVCAR420 and particularly FT190, with the fallopian tube as a potential site of origin for HSGC, has previously not been studied. Here, our assessment of cell viability (at 48 h) in the presence of varying concentrations of G-1 allowed us to determine IC_50_ values of 1.06, 6.95 and 2.58 μM for OV90, OVCAR420 and FT190, respectively. Please also note that a final concentration of 0.5% DMSO was utilized due to the lack of an effect on the cell viability of our studied cell lines (Figure 1) and also other cancer cell lines, e.g., the lung cancer cell line A549 [24] and human leukemia U937 cells [25]. Likewise, earlier studies have suggested that activating GPER inhibits the proliferation of various human cancer cell lines [such as in breast [26], prostate [27] and colorectal cancer [28]. For example, the sustained activation of ERK1/2 through GPER activation has the potential to suppress the growth of colorectal cancer cells in vivo [28]. Using the prostate cancer cell line PC-3, a similar outcome was observed where GPER activation led to the sustained phosphorylation of ERK1/2 levels, ultimately leading to growth arrest [15].

However, the role of GPER in ovarian carcinogenesis remains a matter of controversy [29,30,31]. A previous in vitro study using the ovarian cancer cell lines Caov3 and Caov4 revealed that G-1 treatment led to a significant inhibition of proliferation [15]. Furthermore, activation of GPER through G-1 treatment resulted in sustained levels of p-ERK1/2 and increased H3K4me3 proteins in the Caov3 and Caov4 cells [15]. Sustained activation of ERK1/2 stimulates AP-1 proteins, which in turn triggers the expression of p21. This signaling cascade ultimately results in cell cycle arrest at the G2 checkpoint and, consequently, leads to inhibited cell growth [32]. 

Further, researchers have also demonstrated that treatment with the G-1 agonist activates GPER and results in the suppression of proliferation in another set of ovarian cancer cell lines (SKOV3 and OVCAR3 cells) [5]. The selective GPER agonist G-1 has been shown to suppress the proliferation of SKOV3 and OVCAR3 cells by inhibiting cell cycle progression. The blockade in cell cycle progression has been linked to the increased expression of cyclin B1 and Cdc2, as well as the phosphorylation of histone 3. Furthermore, GPER stimulation leads to a perturbation of mitotic progression, resulting in increased mitotic duration, which triggers caspase-3 cleavage and ultimately leads to cell apoptosis [5]. In ovarian cancer cell lines (IGROV-1 and SKOV-3), G-1 has been found to block tubulin polymerization, which interrupts microtubule assembly and leads to cell cycle arrest in the prophase of mitosis. This results in the suppression of ovarian cancer cell proliferation and apoptosis [9]. These findings suggest that GPER activation via G-1 agonist could inhibit proliferation in case of ovarian cancer [5,15].

Our findings further indicate that G-1 has inhibitory effects on the proliferation of the FT190, OV90 and OVCAR420 cell lines, with OV90 (IC_50_ = 1.06 μM) being relatively the most sensitive and OVCAR420 (IC_50_ = 6.95 μM) being relatively the least sensitive, while FT190 (IC_50_ = 2.58 μM) had a relatively intermediate sensitivity compared to these two other cells lines. These results are consistent with previous studies conducted using ovarian cancer models that also report an inhibition of proliferation [5,15] and might potentially speak to the origin of the OV90 and OVCAR420 cell lines, i.e., it has been suggested that HGSOC, with OV90 and OVCAR420 serving as HGSOC models, originate from either the ovarian surface epithelium or the fallopian tube epithelium (FTE), with the FTE being the more recently suggested site of origin [33]. Currently, OV90 and OVCAR420 are both characterized as HGSOC with no information apparent for their tissue(s) of origin. However, OV90 and FT190 have the more similar IC_50_ values (Figure 2). In addition, untreated OV90 and FT190 cells exhibited a much slower wound-healing rate as compared with untreated OVCAR420 cells (Figure 3). These similarities between OV90 and FT190 could prove useful in guiding future studies exploring the of origin of HGSCO. For example, might OV90 therefore originate from the FTE while OVCAR420 has a different tissue of origin? This is a direction for a future study and currently a question that cannot be answered. Nevertheless, our results are inconsistent with other studies that have reported that GPER promotes growth in the SKOV3 [30] and OVCAR5 [31] cell lines. However, these varying results could be attributable to the utilization of agonists and/or antagonists with distinct specificities, such as estrogen and 1,3-bis(4-hydroxyphenyl)-4-methyl-5-[4-(2-piperidinylethoxy)phenol]-1H-pyrazole dihydrochloride (MPP), in other studies, and as opposed to our utilization of the specific G-1 agonist [15]. In contrast, the G-1 agonist has been identified as a crucial tool for researchers in studying the effects of GPER, owing to its selectivity for this receptor [9,15]. By specifically targeting GPER, the G-1 agonist provides insight into the impact of GPER on cellular behavior [9,15]. Another recent study demonstrated that G-1 treatment inhibited proliferation and induced apoptosis in KGN cells (a human ovarian granulosa cell tumor cell line) in a GPER-independent manner [29]. However, as these results were obtained from a single ovarian cancer cell line, they may not be entirely representative and further investigations will likely be required. 

To investigate whether GPER could influence the migration of the ovarian cancer cell lines OV90, OVCAR420 and FT190, cells were incubated with increasing concentrations of the GPER-specific agonist G-1 for 12 h. Similar studies conducted using in vitro experimental models have also determined that G-1 treatment effectively decreases the migration of the ovarian cancer cell lines Caov3 and Caov4 via GPER activation [15]. Additionally, earlier studies indicate that the activation of GPER inhibits proliferation and migration in diverse human cancer cell lines, including breast [26], prostate [27] and colorectal cancer [28].

## 4. Methods and Materials

### 4.1. Cell Culture and Reagents

Human ovarian cancer cell lines (OVCAR420 and OV90) were obtained from the Drapkin lab at University of Pennsylvania and the American Type Culture Collection (ATCC), respectively. These cell lines are characterized as an ovarian serous adenocarcinoma and a malignant papillary ovarian serous adenocarcinoma, respectively, with both being high-grade serous ovarian cancer cell lines. In addition, an immortalized fallopian tube cell line (FT190) was obtained from the Dana-Farber Cancer Institute (DFCI). The cells were incubated (37 °C) in a humidified atmosphere with 5% CO_2_ and cultured using the recommended medium by the suppliers: Dulbecco’s modified Eagle’s medium/F12 (DMEM/F12; purchased from Hyclone (Logan, UT, USA)) for OVCAR420, OV90 and FT190. The culture medium was enriched with 10% fetal bovine serum (FBS) and an antibiotic-antimycotic solution, which consisted of penicillin (10,000 units/mL), streptomycin (10,000 µg/mL) and amphotericin B (25 µg/mL). Antibiotic-antimycotic and FBS were purchased from Corning (Glendale, AZ, USA) and Gibco (Grand Island, NY, USA), respectively. The day prior to the experiments, the medium was substituted with phenol red-free DMEM (Corning) supplemented with charcoal stripped FBS (Thermo Fisher Scientific; Waltham, MA, USA) and antibiotic-antimycotic. G-1 ((±)-1-[(3aR*,4S*,9bS*)-4-(6-Bromo-1,3-benzodioxol-5-yl)-3a,4,5,9b-tetrahydro-3*H*-cyclopenta[*c*]quinolin-8-yl]-ethanone, ≥98% purity, catalog# 3577) was purchased from Tocris (Bristol, UK). To produce G-1 stock solutions, the chemical was weighed out and slowly added to DMSO while gently vortexing to provide the required stock solution.

### 4.2. Cell Viability Assay

Cells were seeded in phenol red-free DMEM (Corning) supplemented with charcoal stripped FBS and incubated for 24 h. Then, the next day, cells were treated with compound (final DMSO concentration was 0.5%) for 48 h and incubated (37 °C). Finally, cell viability was evaluated using the Cell Titer-Glo Luminescent Cell Viability Assay (Promega; Madison, WI, USA). This assay was employed to quantify the number of viable cells in culture. It measures the presence of adenosine triphosphate (ATP), which serves as an indicator of metabolically active cells. The Cell Titer-Glo Substrate and Cell Titer-Glo Buffer were mixed in equal proportions, and 100 μL of the resulting mixture was added to each well containing 100 μL of growth medium containing cells. Cell lysis was initiated by placing the plates on an orbital shaker for 2 min, followed by a 10 min incubation period for the lysis reactions. Subsequently, ATP levels were quantified using a plate reading luminometer.

### 4.3. Migration Assay (Also Known as a “Wound Healing Assay”)

Cells were seeded in 96-well plates in phenol red-free DMEM (Corning) supplemented with charcoal stripped FBS and incubated for 24 h at 37 °C with 5% CO_2_. Next, a linear wound was created in the cellular monolayer using the IncuCyte Wound Maker tool (Sartorius; Göttingen, Germany), followed by incubation with either treatment or control solution containing 0.5% DMSO for 12 h. The IncuCyte live cell imaging system was used to capture images of the cells after the wound was created. Imaging was conducted over a total duration of 12 h at a magnification of 10×. The IncuCyte^®^S3 program, Scratch Wound analysis pipeline was utilized to calculate the relative wound closure. Percent (%) wound confluence was calculated by means of the following equation [34]:

$$\%wound\;confluence=\left(T0-T12\right)*100/T0$$

T0 refers to the initial open wound area immediately after creating the wound, while T12 represents the open wound area measured 12 h later. 

### 4.4. Statistical Analysis

The mean ± SEM values of at least three separate experiments were performed. GraphPad Prism 9 software was used for data analysis. Differences between groups were analyzed using one-way analysis of variance (ANOVA) with Dunnett’s multiple comparison test by utilizing the GraphPad Prism 9 software (GraphPad Software, Inc., San Diego, CA, USA).

## 5. Conclusions

In conclusion, based on the findings presented in this study, it can be concluded that the GPER agonist G-1 can inhibit cellular proliferation and migration in both the studied fallopian tube and ovarian cancer cell lines. These results indicate that G-1 may have the potential to be developed as an anti-cancer drug.

## Data Availability

The raw data supporting the conclusions of this article will be made available by the authors without undue reservation.
